# Idiopathic nodular glomerulosclerosis: a case report

**DOI:** 10.3389/fmed.2024.1379547

**Published:** 2024-05-20

**Authors:** Claudia Landry, Antonio Rodriguez-Calero, Bruno Vogt, Federica Bocchi

**Affiliations:** ^1^Department of Nephrology and Hypertension, Inselspital, Bern University Hospital, Bern, Switzerland; ^2^Institute of Tissue Medicine and Pathology, University of Bern, Bern, Switzerland

**Keywords:** idiopathic nodular glomerulosclerosis, diabetic nephropathy, nephrotic syndrome, acute kidney injury, chronic kidney disease

## Abstract

Idiopathic nodular glomerulosclerosis (ING) is a rare condition characterized by poor renal prognosis. The pathophysiology remains incompletely understood. Histologically, it closely resembles diabetic nephropathy. The development of this disease seems to be influenced by factors such as metabolic syndrome, particularly hypertension and glucose intolerance, along with active smoking. We report a case of ING in an obese 71-year-old male patient who had a long history of untreated hypertension and smoking. The patient underwent conservative treatment involving the administration of an angiotensin-2 receptor antagonist and dapagliflozin, resulting in favorable disease progression. Additional therapeutic measures, such as discontinuation of smoking and efforts toward weight loss, are strongly advised. Furthermore, regular screening for diabetes in the follow-up is crucial, as it can play a pathophysiological role in the disease and may manifest at a later stage, as observed in our clinical case.

## Introduction

Idiopathic nodular glomerulosclerosis (ING) is a rare disease without clearly distinguishable clinical features, first described by Alpers and Biava (1). The pathological findings encompass the presence of intercapillary nodular glomerulosclerosis, thickening of the glomerular basement membrane, and the occurrence of arteriolonephrosclerosis and hyalinosis, closely resembling the Kimmelstiel-Wilson lesions typically associated with diabetes (2). The exact contribution of factors such as heavy smoking, obesity, and chronic hypertension to the development of the disease is uncertain, thereby posing a diagnostic challenge (3). Clinicians need to remain vigilant regarding this condition, as its prevalence is anticipated to increase alongside the growing incidence of metabolic syndrome. The management approach includes controlling cardiovascular risk factors and initiating anti-proteinuric therapy.

## Case presentation

A 71-year-old Caucasian smoker (60 packs/year), obese (Body Mass Index 48.57 kg/m^2^), male with a history of chronic obstructive pulmonary disease and untreated hypertension was admitted with progressive worsening dyspnea and lower limbs edema over a week. There was no personal history of diabetes mellitus or renal disease.

**FIGURE 1:**
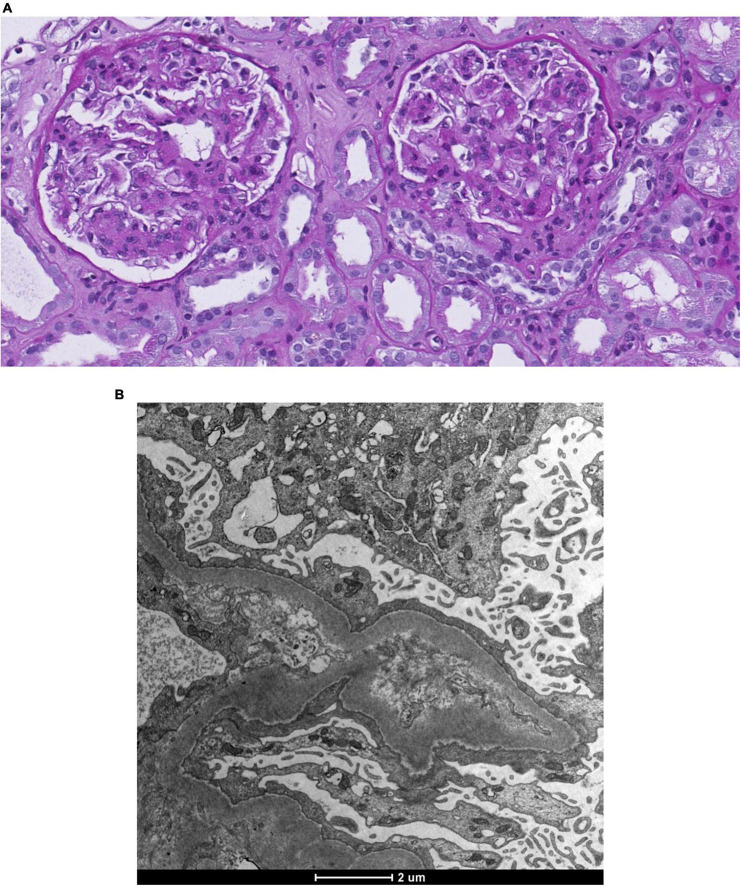
Renal biopsy. **(A)** Light microscopy. Coloration Periodic Acid Schiff. Diffuse nodular expansion of mesangial matrix. **(B)** Electronic microscopy. Basal membrane thickening and foot process effacement.

**FIGURE 2:**
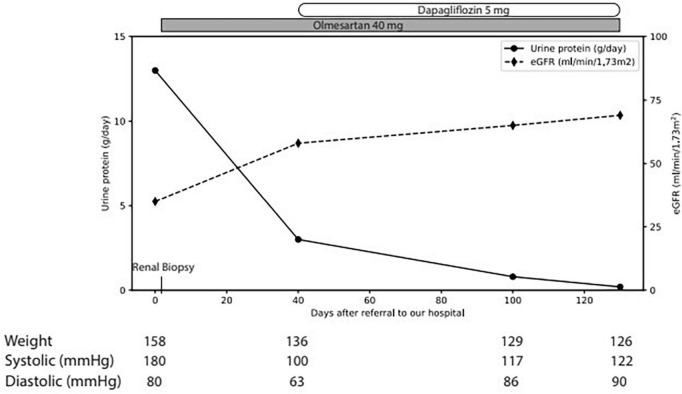
Clinical course. *eGFR* estimated glomerular filtration rate.

The physical examination revealed a blood pressure of 165/65 mmHg, heart rate of 63/min with correct peripheral saturation on room air and a severe anasarca. Laboratory revealed an acute kidney injury (serum creatinine 163 μmol/L, eGFR 36 ml/min), a nephrotic syndrome (proteinuria of 13 g/day, serum albumin 15 g/l [35–52 g/l], triglycerides 3.47 mmol/l [<1.7 mmol/l], LDL-cholesterol 6.28 mmol/l [<3.35 mmol/l]) with a bland urinary sediment. HbA1c excluded diabetes. Immunological markers (anti-nuclear antibodies, antiglomerular basement membrane antibodies, anti-neutrophil cytoplasmic antibodies, anti-dsDNA, anti-histon, anti C1q) and the viral serologies (human immunodeficiency virus, hepatitis B and C) were negative. C3 and C4 and serum protein electrophoresis were normal; immunofixation of immunoglobulins in serum revealed no monoclonal protein. Ultrasound showed normal-sized kidneys with maintained corticomedullary differentiation without obstruction. Percutaneous renal biopsy (Figures 1A, B) revealed 30 glomeruli with diffuse expansion of mesangial matrix and with partial nodular configuration. Additionally, a moderate acute tubular injury was present. Masson trichrome and Congo red stains yielded negative results. Immunofluorescence was negative for immune deposits and electron microscopy showed thickening of glomerular basement membrane (580 nm) and foot process effacement. There was minimal arteriosclerosis and low-grade arteriolohyalinosis. Considering the patient’s medical history, laboratory results, and histological findings, a diagnosis of ING was established. Subsequently, antiproteinuric treatment involving olmesartan and dapagliflozin was initiated, alongside temporary diuretic therapy to restore euvolemia (Figure 2). The patient did not quit smoking and lost 32 kg, initially due to diuretic treatment and later intentionally. Patient follow-up has been favorable, showing blood pressure control; improvement in renal function at 3 months (serum creatinine 95 μmol/L, eGFR 69 ml/min) and resolution of nephrotic syndrome with residual proteinuria of 0.8 g/day.

## Discussion

Idiopathic nodular glomerulosclerosis is an enigmatic and rare glomerular disease with poor prognosis that resembles nodular diabetic glomerulosclerosis. It tends to occur more frequently in older white men, despite the absence of diabetes (2). The incidence of this condition is difficult to ascertain, with only a limited number of prior case series examining both the clinical and histopathologic features. In a recently published cohort (4), the overall incidence of ING was reported to be 0.74%, indicating an enrichment compared to the cohort of Markowitz et al. (3). To ensure the accurate diagnosis of ING, it is crucial to consider other potential differentials that can cause nodular glomerulosclerosis (3). In our case, laboratory results showed impaired glucose tolerance while amyloid nephropathy, membranoproliferative glomerulonephritis, monoclonal immunoglobulin deposition disease and fibrillary and immunotactoid glomerulopathy were ruled out. The observations in this case were deemed consistent with ING. Clinically, similar to other glomerular diseases, the main features of ING’s presentation include the emergence of renal failure accompanied by proteinuria. According to some case series, in up to 40% of cases, the proteinuria can be of nephrotic order (2, 5).

The pathophysiology of ING remains incompletely understood, and there is limited data supporting specific mechanisms underlying the condition. In diabetic patients, nodular glomerulosclerosis is related to the accumulation of advanced glycation end products (AGEs) and extracellular matrix proteins in the mesangial interstitial space (6). This results in cell injury and fibrosis. Others risk factors such long standing hypertension leading to chronic endothelial injury and smoking promoting free radical oxidative stress have been associated with the pathogenesis of ING (3). In a study conducted by Markowich et al. (3) which involved 23 patients diagnosed with ING, a substantial percentage of individuals reported hypertension (95.7%) and smoking (91.3%) as prevalent factors. The findings were validated in a subsequent study by Eadon et al. (4) that included twice as many patients. Moreover, hormonal signaling from obesity, dyslipidemia and insulin resistance have also been discussed (6–8). However, owing to the high and often overlapping prevalence of these risk factors, establishing their causality, rather than a coincidental association, with nodular glomerulosclerosis becomes challenging. Consequently, ING remains a diagnosis of exclusion (see Table 1).

**TABLE 1:** Teaching points.

1	ING is a rare cause of nephrotic syndrome and represent an exclusion diagnosis.
2	Older males are most commonly affected, with associated risk factors including hypertension, heavy smoking, obesity, insulin resistance and dyslipidemia.
3	The primary focus of treatment revolves around smoking cessation and managing high blood pressure with antihypertensive/antiproteinuric therapy.
4	Prognosis remains poor and additional studies are needed to better understand the pathogenesis of this disease.

Histologically, ING findings, especially within the glomeruli, are similar to those of nodular diabetic glomerulosclerosis. Biopsies usually show an increase in the mesangial matrix with nodule formation and thickening of the basement membrane and neovascularization of glomeruli (2, 5). In a study comparing histological findings in ING and diabetic nodular glomerulosclerosis, no significant differences between the two entities were observed in nodular disease severity, degree of glomerular obsolescence, and interstitial fibrosis (2).

Due to the scarcity of information and the absence of prospective trials, the best treatment practices for ING remain unclear. Generally, the treatment approach is primarily conservative, involving smoking cessation and the initiation of antihypertensive/antiproteinuric therapy. The prognosis is generally poor, with approximately 50% of cases progressing to end-stage renal disease 1 year after diagnosis in those who continue to smoke (3).

The clinical case presented here highlighted the intricate interplay of risk factors, including hypertension, smoking, and obesity, in the etiology of this particular lesion pattern. The existence of glucose intolerance in our patient, discovered a few weeks post-discharge, lends support to the hypothesis that there may be an association between ING and heightened sensitivity to glucose levels within the non-diabetic range, leading to an exaggerated glomerulovascular response. Under conservative treatment, the patient’s evolution was favorable, with improvement in renal function and decrease in proteinuria [chronic kidney disease staged G2A3 according to KDIGO guidelines (9)]. Of note was the onset of diabetes 3 months after diagnosis. This underlines the importance for long-term screening for later onset of diabetes. Further studies are essential to better elucidate the pathogenesis of this disease, including the role of impaired glucose metabolism or insulin resistance, as a possible etiology for ING.

## Data availability statement

The original contributions presented in this study are included in this article/supplementary material, further inquiries can be directed to the corresponding author.

## Ethics statement

Written informed consent was obtained from the individual(s) for the publication of any potentially identifiable images or data included in this article.

## Author contributions

CL: Writing – original draft, Writing – review and editing. AR-C: Resources, Writing – original draft. BV: Supervision, Validation, Writing – review and editing. FB: Supervision, Validation, Writing – review and editing.
